# Analysis of Ankle Mobility and Dynamic Valgus Knee in Runners With and Without Patellofemoral Pain Syndrome

**DOI:** 10.1002/hsr2.72051

**Published:** 2026-06-16

**Authors:** Igor Leonardo Alves Mendonça, Flávio Martins do Nascimento Filho, Georgian Badicu, Diego Protásio de Vasconcelos, Tarcísio Brandão Lima, Verena Calmon, Jader Pereira de Farias Neto, Grzegorz K. Jakubiak, Leonardo Yung dos Santos Maciel, Felipe J. Aidar, Walderi Monteiro da Silva Júnior

**Affiliations:** ^1^ Department of Physiotherapy Federal University of Sergipe São Cristóvão Brazil; ^2^ Graduate Program in Health Sciences Federal University of Sergipe São Cristóvão Brazil; ^3^ Department of Physical Education and Special Motricity, Faculty of Physical Education and Mountain Sports Transilvania University of Braşov Braşov Romania; ^4^ Department of Pharmacology, Faculty of Medical Sciences in Zabrze Medical University of Silesia Zabrze Poland; ^5^ CIAFEL, Faculty of Sport University of Porto Porto Portugal; ^6^ Department of Physiotherapy Federal University of Sergipe Lagarto Brazil; ^7^ Federal University of Sergipe São Cristóvão Brazil; ^8^ Department of Physical Education Federal University of Sergipe São Cristóvão Brazil

**Keywords:** knee, patellofemoral pain syndrome, posture, running

## Abstract

**Introduction:**

Patellofemoral pain syndrome (PFPS) is one of the most prevalent musculoskeletal complaints among recreational runners, who commonly engage in various athletic activities. Thus, there is considerable discussion surrounding its risk factors, including dynamic knee valgus (DKV), as well as ankle dorsiflexion restriction (ADR) in a closed kinetic chain. To date, no scientific studies have investigated both ADR and DKV during the execution of the Step‐Down Lateral Test (SDLT) in female runners with and without PFPS.

**Objective:**

To measure ankle dorsiflexion mobility and determine differences in the magnitude and frequency of dynamic knee valgus during the SDLT in recreational female runners with and without patellofemoral pain syndrome.

**Methods:**

This was an analytical, observational, cross‐sectional study involving 40 amateur street runners, aged between 18 and 45 years, divided into a symptomatic group (SG) and asymptomatic group (AG), with allocation performed by a blinded researcher uninvolved in data collection and video analysis. The presence of patellofemoral pain was used to define group allocation and was assessed by an orthopedic surgeon specialized in knee disorders, based on clinical history and physical examination. The outcome measures included the Weight‐Bearing Lunge Test to assess ankle dorsiflexion in a closed kinetic chain, as well as two‐dimensional analysis of dynamic knee valgus during the SDLT. The statistical approach was descriptive and analytical, using the Shapiro–Wilk test, Mann–Whitney *U* test, or Student's *t*‐test, as appropriate.

**Results:**

The final sample consisted of 40 runners, with 22 participants in the AG, while the SG had a total of 18 participants, with homogeneity between groups with respect to age, sex, height and body mass index. The mean DKV in the SG was 17.23° in the symptomatic limb, while in the asymptomatic limb it was 17.35°, from the same group; and in the group without it was 16.01° in the dominant limb. The mean dorsiflexion range of motion for the symptomatic group was 4.40 cm in the symptomatic limb, and 3.99 cm in the asymptomatic limb, while the AG demonstrated a mean of 3.78 cm in the dominant limb. Therefore, no statistically significant differences were observed between groups or between limbs (*p* > 0.05), suggesting that ankle dorsiflexion mobility and dynamic knee valgus during the SDLT do not differ between recreational female runners with and without PFPS.

**Conclusion:**

The findings of this study indicate that there are no significant differences in ankle dorsiflexion mobility or frontal plane knee projection (dynamic valgus) between the studied groups during the SDLT in female recreational runners with and without PFPS.

## 1 Introduction

Patellofemoral pain syndrome (PFPS) is one of the most common complaints among recreational runners [1], accounting for 20%–40% reported knee pain in sports [2]. Epidemiological data show that women are more affected than men [2, 3, 4, 5]). Clinically, PFPS is characterized by anterior knee pain during tasks such as squatting or ascending and descending stairs [4, 6, 7, 8], leading to reduced function and quality of life [6, 9, 10]. PFPS has a multifactorial etiology, and several studies have identified kinetic and kinematic alterations as risk factors at the hip (reduced strength of the external rotators and abductors), knee (reduced extensor strength), and ankle/foot (restricted dorsiflexion range of motion) [6, 11, 12]. The etiology also involves a complex interaction of anatomical, biomechanical, social, psychological, and behavioral factors [12], including increased body mass index, previous injuries, and unsupervised or excessive exercise [13].

Among the biomechanical risk factors, dynamic knee valgus (DKV) and ankle dorsiflexion restriction (ADR) in closed kinetic chain tasks have been studied extensively. Some studies support the association between DKV and PFPS [14, 15, 16], while others do not confirm this relationship [17, 18]. From a biomechanical perspective, DKV during functional tasks may result from both distal and proximal contributions, particularly restricted ankle dorsiflexion [11, 12, 19]. Limited ankle mobility has been linked to reduced knee flexion [20, 21, 22], increased valgus angles [20, 22], and greater ground reaction forces [19, 20, 21, 22, 23, 24, 25, 26]. Notably, individuals with PFPS tend to demonstrate greater medial knee projection during squatting tasks compared to asymptomatic individuals [27, 28]. Mechanistically, limited dorsiflexion constrains forward tibial progression, leading to compensatory strategies such as increased subtalar pronation, tibial internal rotation, hip adduction, and medial knee displacement, which together contribute to greater frontal‐plane knee valgus during squatting and landing tasks [29].

Recent evidence has also highlighted the relevance of inter‐limb asymmetry in lower‐limb biomechanics, suggesting that side‐to‐side differences in joint kinematics and loading may influence patellofemoral joint stress and symptom development. Studies indicate that asymmetrical neuromuscular control and limb loading patterns may be associated with altered movement strategies and increased patellofemoral loading in individuals with knee pain [30, 31]. Therefore, investigating limb‐to‐limb differences may provide a more comprehensive understanding of PFPS‐related biomechanical adaptations.

DKV is commonly assessed using squatting or functional tasks via 2D video analysis, which has shown adequate reliability when compared to 3D motion analysis [32, 33]. A widely used task is the Step‐Down Lateral Test (SDLT) [34], which has shown 85% reliability for detecting DKV in individuals with PFPS [35]. Interestingly, Mansfield et al. [35] reported that medial knee projection during the SDLT was not associated with pain intensity or kinesiophobia. Ankle dorsiflexion mobility is commonly assessed using the Weight‐Bearing Lunge Test (WBLT), a closed kinetic chain assessment with good reliability [36]. Rabin et al. found that reduced ankle dorsiflexion was associated with greater hip adduction and less hip flexion during the SDLT in healthy individuals. However, there is a lack of studies exploring the relationship between ADR and DKV specifically in recreational female runners with and without PFPS.

Only female recreational runners were included in the present study because PFPS presents a higher prevalence and greater biomechanical susceptibility in women, who typically demonstrate greater hip adduction, knee valgus, and altered neuromuscular control during functional tasks when compared to men. Restricting the sample to females reduces sex‐related confounding factors and allows for a more homogeneous investigation of the biomechanical mechanisms associated with PFPS in this high‐risk population.

Considering the clinical importance of PFPS and the biomechanical factors involved, this study aims to contribute to a more accurate understanding of ankle mobility and dynamic knee alignment in this specific population. By focusing on recreational female runners and using functional assessments applicable to clinical settings, this study seeks to address a gap in the literature and help clarify potential biomechanical markers associated with PFPS. Therefore, the primary objective of this study was to analyze whether there are differences in dynamic knee valgus during the Step Down Lateral Test and ankle dorsiflexion range of motion between recreational female runners with and without PFPS. Secondary objectives included: to compare ankle dorsiflexion between symptomatic and asymptomatic groups; to compare ankle dorsiflexion between symptomatic and asymptomatic limbs within the symptomatic group; to analyze limb‐to‐limb differences in DKV within the symptomatic group; and to compare DKV between symptomatic and asymptomatic groups.

## 2 Material and Methods

### 2.1 Study Design

This is an analytical, observational and cross‐sectional study.

### 2.2 Research Location

The survey was carried out at Clínica Live—Orthopedic and Sports Physiotherapy, registered with the CNPJ under number 24.994.959/0001‐58, located at Benjamin Fontes Street, number 198, Luzia, Aracaju, Sergipe.

### 2.3 Sample and Sample Recruitment

**Figure 1:**
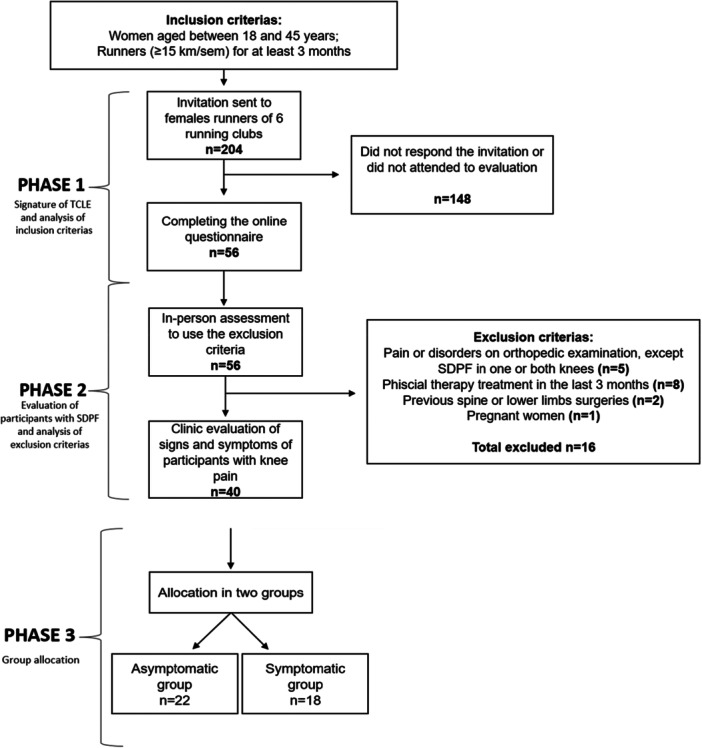
Participant selection process. Abbreviation: PPS, patellofemoral pain syndrome.

The sampling technique was based on the work of Bell et al. [23] and Cheung et al. [9], and they were invited to participate through an official invitation to running clubs, with data being collected from March to May 2021, when comply with the inclusion and exclusion criteria. Thus, sample collection was divided into three phases (Figure 1).

#### 2.3.1 Phase 1

Two hundred and four female street runners from six different running clubs in the city of Aracaju‐Sergipe, Brazil was invited by e‐mail and text messages, then filled out an online questionnaire to analyze the inclusion criteria: between 18 and 45 years old, run at least 16 km per week, using the work ofNoehren, et al. as a basis for this distance, and have been running for at least 3 months. The factor having knee pain or not, was not yet considered in this phase, since the objective of this phase is to consider the inclusion factors only. A total of 56 women street runners answered the first contact and the sociodemographic questionnaire. In the first part of the questionnaire, the informed consent form was signed.

The inclusion criterion requiring a minimum running volume of ≥ 15 km/week was based on studies that consider this threshold representative of recreational runners exposed to a repetitive biomechanical load sufficient to elicit musculoskeletal adaptations and contribute to the development of overuse injuries, such as patellofemoral pain syndrome. Previous evidence indicates that long‐distance runners are more susceptible to overuse‐related injuries, particularly when there is an uncontrolled increase in training load. Therefore, the weekly running volume adopted in this study aims to ensure a sample with meaningful mechanical exposure while remaining within the typical range of recreational practice, thereby enabling the analysis of biomechanical patterns relevant to PFPS.

#### 2.3.2 Phase 2

A face‐to‐face contact was made, with the aim of using the exclusion criteria and making the allocation to the AG or SG. Therefore, in this phase, runners who presented the presence of pain or any other alterations of any origin (congenital, traumatic, metabolic, inflammatory, neurological, or degenerative), genetically inherited or acquired, in the spine or in any of the lower limbs were excluded from the study; previous surgeries on the spine or on any of the lower limbs; pregnant women; or who could not carry out all stages and procedures of the research.

To carry out this exclusion assessment, an assessment was performed by an orthopedic surgeon specializing in knee disorders. Likewise, for the evaluation and confirmation of PFPS, the same surgeon performed an evaluation based on the clinical history and physical examination, without considering imaging tests [6], following the evaluation model described by Yamato et al. [37]. This model is used by the evaluator in the evaluation and diagnosis of PFPS, through some findings, insidious onset pain in the anterior region of the knee, with onset of symptoms at least 3 months, reporting an average of pain on the Visual Analog Scale of pain of 3 (0–10) in the last 3 months, and pain during squatting, sitting for a long period and going up or down stairs. During the clinical examination used to assess the presence of PFPS, specific retropatellar or peripatellar pain on palpation, anterior knee pain when squatting, were used to collaborate in the diagnostic accuracy of PFPS [38].

#### 2.3.3 Phase 3

After identifying symptomatic participants and separated them in two groups (SG and AG), then identified by a badge with a number obtained, which was adjusted to generate a non‐repeating random number (from 0 to 100) for each participant. Only the participant who generated the numbers knew which group the volunteer belonged to, which ensured that the video analysis and tests were not biased, reinforcing the early character of the research.

It is worth mentioning that the member considered dominant was the symptomatic one for the SG group or the one with greater use and dominance in the case of AG.

Initially, 204 female runners from six running clubs were invited to participate in the study (Phase 1). Of these, 148 did not respond to the invitation or did not attend the evaluation, resulting in 56 participants who completed the online questionnaire and proceeded to Phase 2. In Phase 2, all 56 participants underwent an in‐person assessment for the application of the exclusion criteria. A total of 16 participants were excluded due to pain or musculoskeletal disorders identified during the orthopedic examination, except PFPS in one or both knees (*n *= 5), previous physical therapy treatment within the previous 3 months (*n *= 8), previous spine or lower‐limb surgery (*n *= 2), or pregnancy at the time of assessment (*n *= 1). Thus, 40 eligible participants presenting with knee pain advanced to the clinical evaluation of signs and symptoms related to PFPS. In Phase 3, these 40 participants were allocated into two groups according to the presence of symptoms, resulting in an asymptomatic group (*n *= 22) and a symptomatic group (*n *= 18). No dropouts occurred after Phase 2, and all eligible participants completed the subsequent evaluation procedures.

### 2.4 Ethical Aspects

All study procedures were performed in accordance with the standards for research involving human beings (Res. CNS 466/12) of the Council National Health System, respecting ethical standards and the rights of participants. The work was submitted to the Ethics and Research Committee of Universidade Tiradentes via Plataforma Brasil (n°485289). Data were collected with the patients' authorization after signing the Free and Informed Consent Term (FICT), where they received information about the importance of the study and about the procedures to be performed, guaranteeing them all the rights contained in the resolution.

### 2.5 Instruments Used in Data Collection

A questionnaire containing information about the participant's profile and training was used in the research; prepared by all members involved in the study; within this questionnaire, body weight was considered, measured by the DIGI‐HEALTH Serene digital scale, and height, evaluated using a TRADER measuring tape with a maximum capacity of 2 m; the lunge test (weight‐bearing lunge test) was performed to measure the amplitude of dorsiflexion in closed kinetic chain; and finally, for DNV evaluation, during the execution of the step down lateral test, the participant stood on a 20 cm box and with simple reflective markers placed on pre‐established bone structures, which was filmed by a digital camera of a smartphone (iPhone 12; 240 fps; 1080p resolution) [33], supported by a tripod 3 m in front of the box.

### 2.6 Systematics for Data Collection

To standardize the evaluation, an order was followed according to the following stations:

I station: clinical assessment (participant profile questionnaire and anthropometric assessment).
II station: assessment of ankle dorsiflexion mobility.
III station: kinematic evaluation of dynamic valgus during the step‐down lateral test.

#### 2.6.1 Clinical Evaluation

At this station, the volunteer was evaluated using a questionnaire made up, which contains personal data, participant profile and anthropometric data (weight, height, and body mass index). Body mass index (BMI) was calculated as body weight divided by height squared (kg/m^2^). Methodological instruments were used to carry out the anthropometric evaluation, duly tested and calibrated, with a standard error of estimate (SEE) between 2.0% and 3.5% standardized for clinical research. Body weight was assessed using a digital scale DIGI‐HEALTH Serene (Multilaser Industrial, Brazil) maximum capacity of 180 kg. All individuals were instructed to step on the scale barefoot and dress in light clothes, with no accessories in their pockets or other parts of the body. At the time of measurement, they were instructed to distribute the weight of the height that was measured using a stadiometer with a maximum capacity of 2 m and resolution field in millimeters with 5 mm intervals. At the time of the evaluation, the athletes were barefoot, in an orthostatic position with legs and feet parallel, arms relaxed beside the body, palms facing the body and head straight with eyes on the horizon. The individuals' backs were facing the wall, and the measurement was recorded. At the time of measurement, the participant was asked to inhale and hold for 3 s to reduce variations in height.

#### 2.6.2 Assessment of Ankle Dorsiflexion Mobility

**Figure 2:**
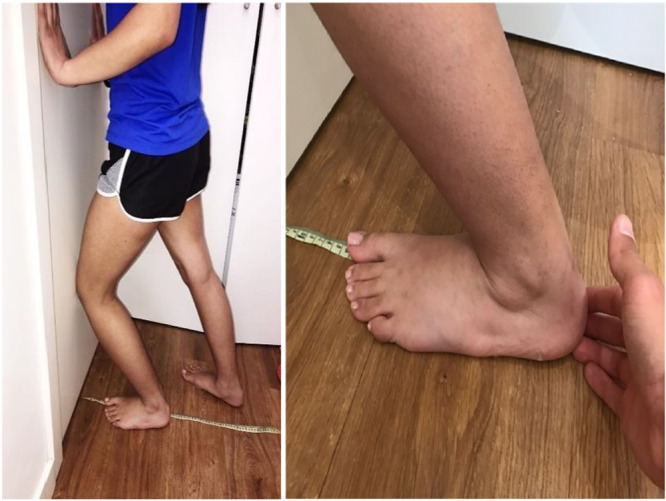
Position of the weight bearing lunge test. *Source:* Research collection.

After removing the shoe, the athlete was directed to a wall without a skirting board, with a marking on the floor represented by a measuring tape cut to just 15 cm. The use of this tape measure is to control and facilitate the maximum point of range of motion. For the initial position, the volunteer was instructed to place the hallux on the tape on the floor, at the 10 cm mark, foot perpendicular to the wall, support both hands on the wall, the contralateral foot could lift the heel, since the only condition of the contralateral limb was that it was also perpendicular to the wall to minimize compensatory effects of the hip. For the execution, the individual will be instructed to touch the knee to the wall without raising the heel of the leg forward, so as not to perform compensatory movements in the ankle (subtalar pronation), knee (valgus or varus) and hip (rotation), internal or external. During execution, to analyze the presence or absence of heel rise, the evaluator places his hand under the heel of the leg being evaluated (Figure 2). The test started with the dominant member, followed by the non‐dominant member.

If in the starting position the athlete manages to touch the knee to the wall without any compensation, the foot was moved away from the wall 1 cm following the tape. If in the starting position or in another position the athlete cannot touch the knee to the wall or perform with compensation, the foot was brought closer to the wall. Once the ideal distance is reached, the achieved distance was considered final. The inter‐clinician reliability is between 0.80 and 0.99, and intra‐clinician is between 0.66 and 0.99 [39]. The weight bearing lunge test measured by the distance between the toe and the wall presented a good correlation with the motion capture angle (*r* = 0.74) [40].

#### 2.6.3 Kinematic Evaluation of Dynamic Valgus During the Step‐Down Lateral Test

**Figure 3:**
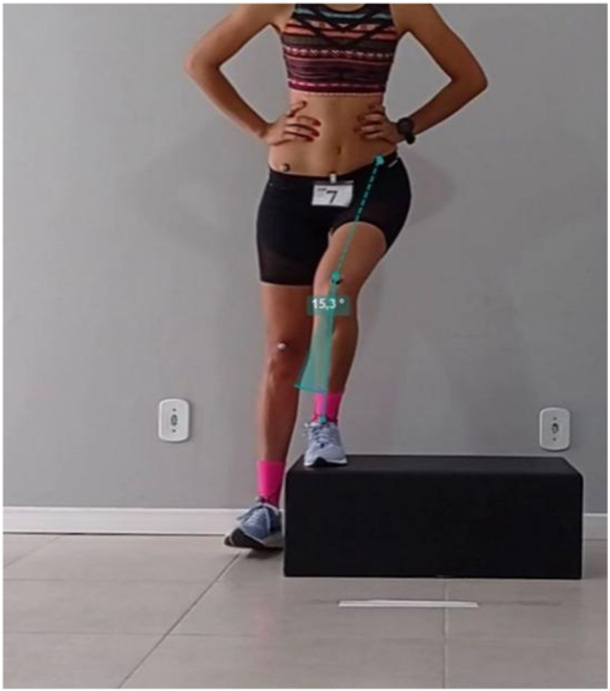
Evaluation of the projection of the knee in the frontal plane during the step downside. *Source:* Research collection.

First, the reflective markers were placed bilaterally in the region of the anterior superior iliac spine, at the base of the patella, and in the region between the malleoli, always by the same evaluator. After video instruction and verbal command, each participant performed a familiarization run and another four sequential repetitions that were considered for data recording and analysis. The execution consists of a single‐leg squat up to approximately 60° of knee flexion, with the foot of the assessed limb close to the lateral edge of the box, arms resting on the waist and the contralateral leg suspended with full knee extension and maximum ankle dorsiflexion, in a step with a height of 20 cm, adjusted according to the height of the participant, so that an amplitude of 60° was reached. The range of motion was controlled by an evaluator using a goniometer, with one arm aligned parallel to the femur and the other parallel to the tibia, with the axis positioned at the lateral joint line. The goniometer was used during the familiarization repetitions and removed during the valid repetitions after ensuring that the participant could independently reach the required range of motion. The distance between the box and the floor was adjusted according to the participant's height using a 5‐cm block placed beside the box to ensure that 60° knee flexion was achieved. In this way, the verbal command was to bring the heel of the contralateral leg closer to the ground, without unbalancing or unloading the weight on the suspended leg and returning to the initial position (Figure 3, Rabin et al. [41]).

Therefore, some considerations were made, and the repetition was canceled and repeated if the volunteer lost balance, rested the contralateral foot on the floor, depositing the body load or removed the arms from the torso. Each repetition should last a total of 5 s, 2 s for the descent, 1 s for the heel strike and 2 s for the return to the original position. Between each recording of a repetition, a 30‐s rest period was taken.

For carrying out the test at the time of the test, three evaluators blinded to the participant's group and dominant or symptomatic limb were trained and recruited, one of them was responsible for placing the reflective markers, another for the verbal instruction and verification of the knee angle, and the last, for the shoot. The evaluator responsible for the recording spoke a sentence to inform which athlete was being evaluated, which repetition and leg was being performed, using a standardized sentence “Athlete X, Step Down Lateral Test, repetition 1, 2 or 3, right or left leg”.

At another time, another blind evaluator, who did not have access to which group an athlete belonged to and which member was being considered, performed the video analysis through the Kinovea application. This application was used to measure the medial projection of the knee during the maximum peak of flexion, in a frontal plane view.

### 2.7 Statistical Analysis

Data was analyzed descriptively and analytically. Categorical variables were presented through their absolute (*n*) and relative (%) frequencies. Numerical variables were tested for normal distribution using the Shapiro‐Wilk test. Parametric data (with normal distribution) were presented using mean (*x*) and standard deviation (SD). Non‐parametric data (without normal distribution) were presented using the median and interquartile range. For the comparison of the medians between the sides, Wilcoxon signed ranks were applied. For intragroup comparisons, the dependent Student's *t*‐test was applied. For comparisons between groups, an independent Student's *t*‐test was applied. Statistical significance was adopted at 5% (*p *≤ 0.05). The statistical program used was IBM SPSS Statistics version 2.

## 3 Results

The evaluation was carried out in 40 amateur street runners, divided into 18 for the SG and 22 for the AG, the presence and absence of (PFPS) was used as a criterion. The SG had a mean age of 35.33 years (±7.85), mean weight of 64.86 kg (±10.87), mean height of 162.89 cm (±7.25) and BMI of 24.42 kg/m² (±3.62). The AG had a mean age of 35.77 years (±6.17), mean weight of 61.55 kg (±7.13), mean height of 162.82 cm (±6.51) and BMI of 23.21 kg/m² (±2.16). The data are presented in Table 1.

**Table 1:** Demographic and clinical characteristics of participants.

Variable	Group		*p* ≤ 0.05
	SG (*n* = 18)	AG (*n* = 22)	
Age, years $\overset{®}{x}$ (DP)	35.33 (7.85)	35.77 (6.17)	0.844
Weight, kg $\overset{®}{x}$ (DP)	64.86 (10.87)	61.55 (7.13)	0.254
Height, cm $\overset{®}{x}$ (DP)	162.89 (7.25)	162.82 (6.51)	0.974
BMI, kg/m^2^ $\overset{®}{x}$ (DP)	24.42 (3.62)	23.21 (2.16)	0.199
Time of practice (months)	54.89 (57.22)	41.14 (29.71)	0.334
Average distance covered per week (km)	20.56 (5.56)	21.00 (6.10)	0.813

*Note:* Values presented as mean (x̅) and standard deviation (SD).

Abbreviations: cm, centimeters; km, kilometers; kg/m², kilograms per square meters.

In addition, the training profile of the participants represents an average time of sports practice of 54.89 months (±57.22) for the symptomatic group and 41.14 (±29.71) for the asymptomatic group.

In the evaluation of the dynamic knee valgus, through the Lateral Step Down, an average of 17.23 (±7.16) was found in the SG and an average of 16.01 (±5.45) in the AG in the dominant limb, as shown in Table 2. Since there was no significant difference between groups, *p* > 0.05 (*p *= 0.543). As well as the mean value of the distance achieved during the lunge test weight‐bearing, to assess ankle mobility, for the SG was 8.83 cm (±3.56), whereas for the AG it was 8. 59 cm (±3.12) in the dominant limb, so there was no significant difference between groups (*p* = 0.820) (Table 2).

**Table 2:** Result of the dynamic valgus angle during the SDL and ankle dorsiflexion mobility during the lunge test weight bearing, comparison between groups. Values presented as mean ± standard deviation. Mann–Whitney and unpaired *t‐*test, *p < 0.05, 95% confidence interval.

Variable	Group		Mean difference	CI 95%	*p* ≤ 0.05
	SG (*n* = 18)	AG (*n* = 22)			
Dynamic knee valgus (degrees)	17.23° (7.16)	16.01° (5.45)	1.22	[−2.81; 5.25]	0.543
Ankle dorsiflexion mobility (cm)	8.83 cm (3.56)	8.59 cm (3.12)	0.24	[−1.78; 2.60]	0.820

*Note:* Values presented as AG (asymptomatic group), centimeters (cm), symptomatic group (SG).

In relation to the symptomatic and non‐symptomatic side in SG runners when faced with dynamic valgus, there was no significant difference between limbs (*p* = 0.956). The average for the painful limb was 17.23° (±7.16) and for the non‐painful limb 17.35° (±5.13). Furthermore, in relation to ankle mobility between the symptomatic limb, mean of 8.83 cm (±3.56), and the asymptomatic limb, mean of 9.28 cm (±2.98), there was no significant difference between the legs (*p *= 0.119) (Table 3).

**Table 3:** Result of dynamic valgus angle during SDL and ankle dorsiflexion mobility during lunge test weight bearing, comparison between symptomatic and asymptomatic limbs.

Variable	SG lower limb analyzed		Mean difference	CI 95%	*p* ≤ 0,05
	Symptomatic (*n* = 18)	Asymptomatic (*n* = 18)			
Dynamic knee valgus (degrees)	17.23° (7.16)	17.35° (5.13)	−0.12	[−4.33; 4.10]	0.956
Ankle dorsiflexion mobility (cm)	8.83 cm (3.56)	9.28 cm (2.98)	−0.44	[−1.90; 2.38]	0.119

*Note:* Values presented as centimeters (cm). Values presented as mean ± standard deviation. Mann–Whitney and unpaired *t‐*test.

*p < 0.05, 95% confidence interval.

## 4 Discussion

This study aimed to determine whether ankle dorsiflexion range of motion is associated with dynamic knee valgus during the lateral step‐down Test in female recreational runners with and without patellofemoral pain syndrome (PFPS). Contrary to expectations derived from prior literature, no significant differences were observed between symptomatic and asymptomatic groups in either ankle dorsiflexion mobility or dynamic knee valgus angle.

These findings suggest that dorsiflexion range of motion may not constitute a primary determinant of frontal‐plane knee kinematics during tasks of moderate neuromechanical demand. Previous studies have associated restricted dorsiflexion with increased dynamic valgus in high‐impact tasks such as jump‐landing and drop jumps [22], which involve greater ground reaction forces and complex neuromuscular coordination. In contrast, the lateral step‐down test might not sufficiently challenge the neuromuscular system to expose subtle compensatory patterns in runners with PFPS, thereby limiting its sensitivity for detecting clinically relevant kinematic deviations.

The absence of group differences reinforces the conceptualization of dynamic knee valgus as a multifactorial phenomenon arising from the interaction of neuromuscular, proprioceptive, and biomechanical factors [42]. This interpretation aligns with motor control theories emphasizing task‐oriented organization of movement rather than isolated joint function [43]. Consequently, the relationship between local joint mobility and global movement coordination should be interpreted within the broader context of sensorimotor integration and task‐specific motor strategies.

An additional methodological consideration relates to the nature of verbal instructions provided during the test. Participants received internally focused cues (i.e., directing attention toward body segments), which may have constrained automatic motor control processes. Evidence suggests that externally focused instructions—directing attention toward environmental outcomes—can facilitate movement efficiency and enhance lower‐limb alignment [44]. This procedural aspect may have contributed to the lack of observable intergroup differences by promoting a more uniform strategy among participants.

The relatively small sample size represents a further limitation, potentially reducing the statistical power to detect small‐to‐moderate effects. Moreover, the present analysis relied on two‐dimensional kinematic data, which may not adequately capture multi‐planar interactions underlying dynamic valgus. Future investigations should employ three‐dimensional motion capture systems to enable a more comprehensive assessment of lower‐limb kinematics and intersegmental coordination.

Advances in pattern recognition and machine learning techniques have demonstrated considerable potential for identifying complex, non‐linear movement patterns in gait and sports biomechanics [45]. Incorporating such computational frameworks could enhance the discriminatory capacity of kinematic analyses and facilitate the identification of distinct movement signatures associated with PFPS.

Furthermore, the inclusion of more detailed quantitative data and visual representations—such as joint angle distributions, 3D trajectory plots, and statistical parametric mapping—would improve result transparency and interpretability. Such graphical and data‐driven approaches could also assist clinicians and researchers in recognizing subtle alterations in motor behavior not readily apparent through traditional summary metrics.

In summary, the present findings highlight the limitations of relying solely on static or single‐joint measures such as ankle dorsiflexion range of motion to infer dynamic movement performance. Comprehensive evaluation protocols integrating three‐dimensional kinematic analysis, neuromuscular control assessment, and advanced computational modeling are warranted to more accurately characterize the movement adaptations associated with PFPS. This integrative approach may ultimately contribute to more effective diagnostic and rehabilitation strategies grounded in individualized motor pattern recognition.

## 5 Conclusion

Clinicians should be cautious when interpreting ankle dorsiflexion range of motion as an isolated contributor to dynamic knee valgus during the Lateral Step‐Down Test in female recreational runners with PFPS. The present findings suggest that moderate‐demand tasks such as the SDLT may not be sufficiently sensitive to detect subtle kinematic differences between symptomatic and asymptomatic individuals. Therefore, clinical assessment should combine multiple functional tasks with varying neuromechanical demands and consider task instructions and movement strategies when evaluating frontal‐plane knee control.

## Author Contributions

Conceptualization by I.L.A.M. and F.M.d.N.F. Methodology by F.J.A. and G.B. Software by D.P.d.V. and T.B.L. Validation by V.C., J.P.d.F.N., and G.K.J. Formal analysis by I.L.A.M., F.J.A., G.B., and V.C. Investigation by J.P.d.F.N, L.Y.d.S.M, ad W.M.d.S.J. Resources by G.K.J. Writing – original draft preparation by I.L.AM. and F.M.d.N.F. Writing – review and editing by G.B., I.L.AM., and F.M.d.N.F. Visualization by I.L.A.M. and F.M.d.N.F. Supervision by W.M.d.S.J.

## Funding

The authors received no specific funding for this work.

## Consent

Any informed consent was obtained from all subjects involved in the study.

## Conflicts of Interest

The authors declare no conflicts of interest.

## Transparency Statement

The corresponding author, Georgian Badicu, affirms that this manuscript is an honest, accurate, and transparent account of the study being reported; that no important aspects of the study have been omitted; and that any discrepancies from the study as planned (and, if relevant, registered) have been explained.

## Data Availability

The data sets generated during and/or analyzed during the current research are available from the corresponding authors upon reasonable request.
